# Externalizing psychopathology and persistence of offending in childhood first-time arrestees

**DOI:** 10.1007/s00787-012-0257-x

**Published:** 2012-02-24

**Authors:** Moran Cohn, Lieke van Domburgh, Robert Vermeiren, Charlotte Geluk, Theo Doreleijers

**Affiliations:** 1 Department of Child and Adolescent Psychiatry, VU University Medical Center Amsterdam, P.O. Box 303, 1115ZG Duivendrecht, The Netherlands; 2 LSG-Rentray, Zutphen, The Netherlands; 3 Department of Child and Adolescent Psychiatry, Curium-LUMC, Leiden, The Netherlands; 4 Medicines Evaluation Board, Den Haag, The Netherlands; 5 Department of Criminal Justice, Law Faculty, Leiden University, Leiden, The Netherlands

**Keywords:** Attention deficit/hyperactivity disorder, Oppositional defiant disorder, Conduct disorder, Delinquency, Forensic, Prevention/early identification

## Abstract

This study aims to investigate the predictive validity of externalizing psychopathology for persistence in delinquent behavior when controlling for socio-demographic and first arrest characteristics in childhood first-time arrestees. A sample of first-time arrestees aged under 12 (*n* = 192) was assessed using the Diagnostic Interview Schedule for Children (DISC-IV) parent-version on attention deficit/hyperactivity disorder (ADHD), oppositional defiant disorder (ODD) and conduct disorder (CD). Based on child and parent reports of offending as obtained at arrest and at 2-year follow-up, three groups of offenders were differentiated: (1) persistent high (*n* = 48), (2) occasional (*n* = 62), and (3) persistent low offenders (*n* = 82). Over one-third of the sample (33.9%) was diagnosed with an externalizing disorder, and 13.5% with both ADHD and ODD or CD. Higher levels of externalizing psychopathology distinguished persistent high offenders from occasional (comorbid ADHD and ODD/CD: OR 8.2, CI 2.6–25.5) and persistent low offenders (comorbid ADHD and ODD/CD: OR 18.2, CI 4.6–72.3; ADHD: OR 4.1, CI 1.3–13.0), over and above socio-demographic and first offense characteristics. Living with both biological parents distinguished the persistent low offenders from the occasional offenders (OR 2.5, CI 1.2–5.0). Since the prevalence of externalizing disorders was high and predicted re-offending, mental health screening and intervention initiatives, aiming at these conditions, should be investigated for this high-risk sample.

## Introduction

Juveniles who display delinquent behavior prior to adolescence are two to three times more likely to become chronic violent offenders compared to those with a later onset [1]. A first police contact during childhood is a particular strong predictor of subsequent serious [2] and persistent delinquency [3, 4]. However, earlier research has shown that a substantial subgroup of childhood delinquents does not persist in offending [5–7]. Therefore, identifying individuals at risk for continuous offending carries substantial relevance, as it may enable focusing scarce resources on those most in need, i.e., by targeted use of prevention and intervention initiatives [8, 9].

At present, studies on predictors of persistence among childhood onset offenders have been scarce, in contrast to an abundance of studies on older groups, i.e., adolescent and adult offenders [10]. Over the last few decades, a number of population-based studies have focused on risk factors of delinquency [11–14]. However, childhood onset offenders are scarce and thus constitute only a small group within these population-based studies, limiting the possibility to investigate differences within this specific subgroup [15]. As a result, it is not known whether risk factors related to the onset of offending as identified in the general population bear value for predicting persistence among childhood offenders. Therefore, this study will focus on such determinants within a group of childhood first-time police arrestees.^1^

The presence of oppositional defiant disorder (ODD) or conduct disorder (CD) is likely to be a predictor of persistence in offending, as both diagnoses reflect stabile behavioral patterns. In general population studies, ODD and CD have been identified as strong predictors of serious and persistent offending and antisocial behavior [16]. Until now, no studies have investigated whether the more prevalent diagnosis ODD predicts persistence in very young offender populations. CD, however, has been found to predict recidivism in adolescent offender populations [17, 18], over and above offense characteristics [19]. Early identification of externalizing disorders in childhood first-time police arrestees may, therefore, be of special interest. Because, official offending history is by definition lacking in this population, such diagnoses may be important predictors of persistence of offending. Moreover, research suggests that evidence-based interventions available for these disorders are likely to be more effective in childhood than later in life [20], thus possibly reducing risk of persistence and negative outcome.

Moffitt [21] previously indicated that hyperactivity, inattention and impulsivity are characteristic of the subgroup of life course persistent (LCP) offenders. Findings on the predictive value of attention deficit/hyperactivity disorder (ADHD) for subsequent offending within offender subgroups have, however, so far been inconclusive. While some have reported a positive relation between ADHD and offending [22], others have not found such a relationship [23]. Still, others found that ADHD was not predictive of re-offending, while the comorbidity of ODD/CD and ADHD was [24–26]. Because, comorbidity of ADHD and externalizing disorders is more prevalent in childhood than in adolescence, this condition may even constitute an age-specific determinant [27, 28]. Therefore, our study will focus specifically on the predictive validity of ADHD in relation to other diagnoses.

For reasons mentioned above, the aim of the current study was twofold. First, to investigate the prevalence of externalizing psychiatric disorders (ADHD, ODD and CD) in a group of childhood first-time police arrestees. Second, to study the 2-year predictive validity of externalizing disorders, socio-demographic, family, and offense characteristics for self-reported delinquency. It is hypothesized that the prevalence of psychopathology in this sample will be higher than in the general population. Further, it is hypothesized that externalizing disorders, and in specific comorbidity of ADHD and ODD/CD, will predict recidivism at follow-up over and above socio-demographic and first-time arrest characteristics.

## Method

### Sample

The sample consisted of 192 first-time arrestees with a mean age of 10.3 years (SD 1.5, range 5–12) who had been arrested and assessed in the period July 2003 to December 2005 and re-assessed 2 years later. The majority (86.5%) was male. About half (57.1%) of the sample was native Dutch, while 11.0% was of Moroccan, 8.9% of Antillean (Dutch Caribbean), 6.8% of Turkish, and 16.2% of another ethnic origin. Nearly half (46.8%) of the children lived in a neighborhood of low socio-economic status (SES) [29].

### Procedure

The study was approved by the VU University Medical Ethics Committee and the Ministry of Justice and has therefore been performed in accordance with the ethical standards laid down in the 1964 Declaration of Helsinki. Subjects were selected from local police registration systems of three police districts in The Netherlands to assure sufficient variability in SES and levels of urbanization of the neighborhoods the children resided in. Offending was defined as behavior that could be prosecuted if displayed by someone aged 12 years or older, excluding status offenses. Researchers gave oral and written information about the study and obtained written informed consent from both children and parents before starting the study. At 2-year follow-up, participants were re-assessed. The mean period between the first measurement and follow up was 2.24 (SD 0.36) years. Overall, 73.0% (*n* = 308) of the children who were referred to the researchers (*n* = 422) by the police participated in this study. Children who refused participation did not differ from participants on age and seriousness of first arrest, but were more often female (21.1 vs. 12.7%; *χ* ^2^ = 4.55, df = 1, *p* = 0.033), of non-Dutch origin (65.8 vs. 51.0%; *χ* ^2^ = 7.17, df = 1, *p* = 0.007) and more often lived in neighborhoods with low SES (68.4 vs. 52.6%; *χ* ^2^ = 8.49, df = 1, *p* = 0.004). Of these 308 participants, 62 did not participate at follow-up while another 54 were excluded (mostly because of missing data on the diagnostic interview, due to language problems), resulting in a final sample of 192 children. Excluded children and non-participants at follow-up did not differ from included children on gender, age of first arrest, or seriousness of offense leading to arrest. However, excluded children and non-participants at follow-up were more often of non-Western origin (*χ* ^2^ = 27.6, df = 2, *p* < 0.001) and resided in neighborhoods of lower SES (*χ* ^2^ = 5.2, df = 1, *p* = 0.022).

### Dependent variable

#### Self-reported level of offending

Level of offending was based on the Observed Antisocial Behavior Questionnaire (OAB) [30]. The OAB is an age-appropriate adaptation of the self-report of antisocial behavior [31] and investigates antisocial behavior over the previous half year at T0 and 1 year at follow-up. Items on offending were summed to create a level of offending (range 0–17). The score was based on the following 17 items: (1) stealing outside the home (five items); (2) hitting or fighting outside the home (five); (3) property damage and arson (four items); (4) rule breaking and fare dodging (two items), and (5) weapon possession (one item). Both child self-report and parent report versions were combined, and an item was coded present if one of the informants reported the behavior.

### Independent variables

#### Socio-demographics and family characteristics

A structured checklist [32] was used to assess the following characteristics: gender, age of mother at first birth, ethnicity, previous police-contacts for offending by family members and information about the SES of the neighborhood in which the child lived. Parental mental health problems were investigated with the Symptom Checklist SCL-90 [33, 34] and four questions concerning the presence of psychological or psychiatric problems, alcohol abuse or drug use in one of the parents [32]. If either one or both of the parents scored affirmatively on at least one of the items of the checklist and/or in the clinical range of the SCL-90, the variable was considered to be present.

#### Official first offense characteristics

Official delinquency was derived from police database systems. To assign a level of seriousness to the first offense as registered by the police, a classification of Seriousness Of Early Police Registration (SEPR) was developed. The SEPR is an adaptation of the General Level of Seriousness Classification as developed by Loeber et al. [35]. Offense seriousness was dichotomized as follows: (1) minor delinquency (e.g., minor verbal aggression, shoplifting, minor vandalism) and (2) moderate to severe delinquency (e.g., theft, serious arson, sex offenses, robbery).

#### Externalizing disorders

The parent version of the National Institute of Mental Health (NIMH) Diagnostic Interview Schedule For Children (DISC), version IV ([36], Dutch translation: [37]), was used to diagnose ADHD, ODD and CD. A diagnosis of ADHD was assigned if the child met diagnostic criteria for the inattentive, the hyperactive-impulsive or the combined type. Since ODD and CD are highly interrelated [38], and because, CD occurs infrequently and mostly in a mild form at such a young age, subjects who scored either or both of these diagnoses were classified as having ODD/CD.

### Analyses

For statistical analysis, the Statistical Package for Social Sciences (SPSS 12.0.1) was used. For all calculations *α* was set at 0.05. First, subgroups were created based on the stability of self- and parent-reported delinquency over the 2-year follow-up period: (1) children who scored above the 75th percentile, both initially and at follow-up on the child and/or parent version of the OAB, were considered to be persistent high offenders; (2) children who scored high on only one of the assessments were named occasional offenders, and (3) children who scored below the 75th percentile twice were considered persistent low offenders. Second, externalizing psychopathology, socio-demographics and offense characteristics were compared using means for continuous and percentages for categorical variables, and between-group differences were calculated using *χ* ^2^-tests for the categorical and Student’s *t*-tests for the continuous variables. Because, CD was expected to occur at a low rate in this child sample, ODD and CD were taken together and compared as a combined ODD/CD variable. Third, to predict offending group membership, three hierarchical logistic regression analyses were performed, one for each group contrast (persistent low vs. persistent high, occasional vs. persistent high, persistent low vs. occasional) using forward selection. Characteristics that differentiated between groups in the bivariate analyses at *p* < 0.1 were entered as independents in two blocks, (1) socio-demographic, family and offense characteristics, and (2) externalizing psychopathology,^2^,^3^ To gain insight into the predictive accuracy of the regression models, false positives and false negatives will be reported on. Because, the aim was to study the outcome in relation to unique individual disorders and co-morbidity, diagnoses were studied as a combined categorical variable (no disorder, ADHD, ODD/CD, ADHD and ODD/CD). In the regression models, this categorical variable was recoded into three dummy-variables. With ‘no disorder’ as reference category, the dummy-variables were (1) ADHD-only, (2) ODD/CD-only and (3) co-morbid ADHD and ODD/CD. Given the small number of participants in each subgroup we were not able to study interaction effects.

## Results

### Prevalence of self-reported offending

Based on child and parent reports, 25.0% of the participants scored high levels of offending on both assessments and were thus classified as persistent high offenders. Nearly one-third (32.3%) reported high levels of offending on one of the assessments, and were thus classified as occasional offenders, while 42.7% reported low levels of offending at both assessments. Table 1 shows the number of offenses both initially and at follow-up for all three offender groups. Groups differed significantly in the number of offenses committed at each assessment.

**Table 1:** Offense rates in offending subgroups

	High (*n* = 48) mean (SD)	Occasional (*n* = 62) mean (SD)	Low (*n* = 82) mean (SD)	All (*n* = 192) mean (SD)	Between-group comparisons		
					High versus occasional	High versus low	Occasional versus low
Initial offense rate	3.9 (2.0)	2.1 (1.7)	0.5 (0.5)	1.9 (2.0)	***	***	***
Follow-up offense rate	4.5 (2.7)	1.6 (1.7)	0.2 (0.4)	1.7 (2.4)	***	***	***

Student’s *t*-tests were used to compare offense rates between offending subgroups

*** *p* < 0.001

### Prevalence of externalizing psychiatric disorders

Table 2 shows the prevalence rates of ADHD, ODD/CD and comorbid ADHD and ODD/CD. Over one-third of the first-time arrestees had an externalizing disorder, while 13.5% was diagnosed with co-morbid ADHD and ODD/CD. Regarding group differences, externalizing disorders, in general, were more common in the persistent high group compared to other groups, and higher in the occasional offender group compared to the persistent low group. With respect to specific diagnoses, ADHD-only did not differ between offender groups, while there was a trend toward higher prevalence of ODD/CD-only in the occasional group than in the low group. Co-morbid ADHD and ODD/CD was significantly more common in the persistent high group than in the persistent low and the occasional group.

**Table 2:** Rates of externalizing disorders in offending subgroups

	High (*n* = 48) %	Occasional (*n* = 62) %	Low (*n* = 82) %	All (*n* = 192) %	Between-group comparisons *χ* ^2^(df = 1), *p*		
					High versus occasional	High versus low	Occasional versus low
Any externalizing disorder	62.5	33.9	17.1	33.9	8.917**	27.904***	5.415*
ADHD-only	16.7	12.9	8.5	12.0	–	–	–
ODD/CD-only	8.3	12.9	4.9	8.3	–	–	2.977^†^
ADHD and ODD/CD	37.5	8.1	3.7	13.5	14.175***	25.601***	–

Omnibus test for externalizing disorders: χ^2^ 41.5 (df = 6) *p* < 0.001. Non significant values are denoted by –

^†^0.1 < *p*<0.05, * *p* < 0.05, ** *p* < 0.01, *** *p* < 0.001

### Socio-demographic, neighborhood, and offense characteristics

As Table 3 shows, the group of childhood first-time police arrestees as a whole is characterized by high prevalence of both socio-demographic and family risk-factors. However, few differences in socio-demographic and family characteristics were found between the different offender groups. Children from the persistent high and occasional group more often came from broken families compared to those from the persistent low group, and their parents had higher rates of mental health problems. Compared to the persistent low group, children in the occasional offender group were more often of non-Dutch ethnicity. Arrest of family members was reported more often in the occasional group than in the low group. First-time official offenses were mostly of moderate severity. First-time arrests of children in the persistent low group were more often of low severity than first-time arrests of children in the occasional group.

**Table 3:** Rates of socio-demographic, family and offense characteristics in offending subgroups

	High (*n* = 48) %	Occasional (*n* = 62) %	Low (*n* = 82) %	Between-group comparisons *χ* ^*2*^(df = 1), *p*		
				High versus occasional	High versus low	Occasional versus low
Low SES neighborhood	63.8	64.5	61.7	–	–	–
Family arrests	46.8	46.7	32.5	–	–	2.904, 0.088
Parental psychiatric disorder	37.5	37.1	22.0	–	3.656, 0.056	3.977, 0.046
Not living with both parents	58.3	56.5	32.9	–	8.007, 0.005	7.969, 0.005
Teenage motherhood	12.8	17.7	13.8	–	–	–
Non-Dutch ethnicity	40.4	51.6	37.8	–	–	2.735, 0.098
Moderate to serious first arrests	44.7	58.1	41.5	–	–	3.895, 0.048
Age first-arrest^a^	10.8 (1.2)	10.7 (1.7)	10.5 (1.5)	–	–	–

Chi-square tests were run, except for “Age first-arrest” in which Student’s *t*-test was used to test for differences

^a^The values are given in mean (SD)

### Prediction of persistence

To predict persistence in offending, we performed three logistic regression analyses, one for each group comparison (i.e., persistent high vs. occasional, persistent high vs. persistent low, occasional vs. persistent low).

#### Persistent high versus occasional offenders

No differences in socio-demographic, offense or family characteristics where found between persistent high and occasional offenders in bivariate analyses, therefore only externalizing disorders were entered into the regression model. Co-morbid ADHD and ODD/CD significantly predicted being in the persistent high compared to the occasional group, accurately classifying 68% of the children (true-positives 67%, true-negatives 69%) and explaining 19% of variance (Table 4).

**Table 4:** Prediction of persistent high versus occasional offending

Overall model: *χ* ^2^ = 16.581, df = 3, Nagelkerke *R* ^2^ = 0.19	*B*	Wald	POR (95% CI)	*p* value
No disorder (ref)		14.0		0.003
ADHD-only (dummy 1)	0.82	2.05	2.3 (0.7–7.0)	0.15
ODD/CD-only (dummy 2)	0.13	0.04	1.1 (0.3–4.3)	0.85
ADHD and ODD/CD (dummy 3)	2.10	13.2	8.2 (2.6–25.5)	<0.001

*ref* The group with no diagnosis is the reference category, *POR* partial odds ratio

#### Persistent high versus persistent low offenders

To predict the persistent high versus low offending, parental mental health problems and not living with both biological parents were entered at step 1, and externalizing psychopathology was entered in step 2. In step 1, not living with both biological parents distinguished between persistent high and low offenders. After entering externalizing psychopathology in step 2, only ADHD and co-morbid ADHD and ODD/CD significantly predicted being in the high group compared to the persistent low group. Family factors no longer contributed significantly to the prediction model. The model explained 32% of the variance (Table 5), and accurately classified 72% of the persistent high versus low offenders (true-positives 69%, true-negatives 72%).

**Table 5:** Prediction of persistent high versus persistent low offending

Overall model: *χ* ^2^ = 35.022, df = 4, Nagelkerke *R* ^2^ = 0.32	*B*	Wald	POR (95% CI)	*p* value
Step 1				
Lives with biological parents	1.05	7.79	2.9 (1.4–6.0)	0.005
Step 2				
Lives with biological parents	0.47	1.10	1.6 (0.7–3.9)	0.30
No disorder (ref)		21.5		<0.001
ADHD-only (dummy 1)	1.42	5.86	4.1 (1.3–13.0)	0.016
ODD/CD-only (dummy 2)	1.44	3.54	4.2 (0.94–19.1)	0.06
ADHD and ODD/CD (dummy 3)	2.90	16.9	18.2 (4.6–72.3)	<0.001

*ref* The group with no diagnosis is the reference category, *POR* partial odds ratio

#### Occasional versus persistent low offenders

To distinguish between occasional and low offenders, family arrests, parental mental health problems, not living with both biological parents, ethnicity and first-time offense severity were entered at step 1. However, only not living with both biological parents was selected in the forward selection procedure. In the second step, externalizing psychiatric disorders were entered but not selected, as they did not add significantly to the prediction model. Not living with both biological parents accurately predicted being in the occasional versus the persistent low offender group in 62% of the cases (true-positives 56%, true-negatives 66%) and explained 6.4% of the variance (Table 6).

**Table 6:** Prediction of occasional versus persistent low offending

Overall model: *χ* = 6.833, df = 1, Nagelkerke *R* ^2^ = 0.064	*B*	Wald	POR (95% CI)	*p* value
Lives with biological parents	0.91	6.68	2.5 (1.2–5.0)	0.01

*POR* Partial odds ratio

## Discussion

The current study aimed to investigate the prevalence of externalizing psychiatric disorders (ODD, CD, or ADHD) in childhood arrestees and to study the predictive value of these disorders for persistence of self-reported offending at 2-year follow-up. Persistent offending was shown by a small but substantial subgroup, with 25% scoring high on self-reported offending both initially and at follow-up. In contrast, as much as half of the group scored low persistently. Remarkable high rates of externalizing disorders were found, as over one-third of the sample was diagnosed with at least one externalizing disorder, and 13.5% with comorbid ADHD and ODD/CD. In a combined regression model including socio-demographic, familial and criminological first arrest characteristics, only comorbid ADHD and ODD/CD significantly predicted persistent high offending as compared to occasional offending. Persistent high offending compared to low offending was also predicted by comorbid ADHD and ODD/CD and, to a lesser extent, by ADHD-only. Not living with both biological parents predicted occasional versus low offending. Our findings emphasize the relevance of identifying mental health problems among childhood first-time arrestees, as these disorders may relate to negative outcome and are treatable by means of evidence-based interventions.

While childhood first-time arrestees are at higher risk to persist, this study shows that by far not all actually continue on a delinquent path. This indicates that not all childhood arrestees belong to the early onset and life-course persistent group as defined in Moffitt’s [14] original developmental model. Based on our findings, mental health assessment may support the accurate identification of children who are likely to continue. In this study, follow-up period was relatively short. Some children may start re-offending later on, or may desist shortly afterwards [5, 6]. However, most studies show that re-offending occurs most frequently within the first 2 years [39]. Our findings indicate that childhood arrestees should be considered an at-risk group, but similarly, that a substantial group does not persist in deviant behavior.

As expected, prevalence rates of externalizing disorders were much higher than those in the general Dutch population (8%) [37], as one-third of the first-time arrestees met the criteria for ADHD, ODD and/or CD. The combination of ADHD and ODD/CD was found to be particularly predictive of persistently high levels of offending, while ODD/CD-only was not (although there was a trend). Low predictive validity of ODD/CD-only in our study may have been caused by the large proportion of ODD cases. Oppositional behavior as measured by ODD is less specifically related to delinquent development, as it may also reflect other pathological developmental patterns, such as anxiety or depression [40]. Future studies should incorporate larger ODD and CD subgroups, to make it feasible to study their predictive validity for persistence of offending separately. Conduct problems in general are known to develop as a result of temperamental predisposition and environmental factors (for review, see [41]) and can therefore increase and decline in unison with temporary environmental influences (such as negative peer-associations). Possibly, conduct problems with comorbid ADHD are more persistent, because, they are to a higher degree subject to a stable neurobiological deficit, related to the latter, than conduct problems without ADHD. Because, externalizing disorders did not distinguish occasional from persistent low offenders, it may be suggested that the persistent high offending subgroup, in specific, constitutes a qualitatively different group compared to others. This would be in line with findings from Moffitt [14], who distinguishes a life course persistent subgroup marked by persistent conduct problems, ADHD and early onset of ODD/CD. The high level of psychiatric disorders in this sample stresses the need for their detection and treatment, the more so as they are predictive of persistence in offending.

Contrary to some previous studies [26], but in line with others [22], ADHD-only differentiated between the persistent high and low offending group in our sample, after taking into account the influence of family characteristics. First, although our sample was not large enough to test for their specific interactions, this finding demonstrates the necessity to take into account confounding effects of environmental factors, when studying the influence of psychiatric disorders. Second, as mentioned above, the predictive value of ADHD for persistence in our sample of early-onset offenders is in line with Moffitt’s description of early-onset life course persistent offenders [14]. Furthermore, while it remains unclear whether ADHD predicts persistent offending in non-offender samples [22, 26], these findings confirm, in line with Lahey and Loeber [42], that ADHD is a moderator of behavioral continuity in persons already on the antisocial path.

In line with the previous findings [43], several easy-to-register socio-demographic and offense characteristics were only of limited value for predicting re-offending among this high-risk sample. As these first-offenders by definition do not have official histories of offending, offense characteristics seem to lack the power to distinguish those at risk of re-offending. Similarly, rather crude socio-demographic risk factors as identified in the general population do not seem to distinguish those most at risk in high-risk samples, although they should be taken into account as possible confounding factors, as mentioned above. Therefore, a first arrest below 12 may be a valuable sign in the detection of high-risk children, but in-depth assessment is needed to distinguish who is at risk and who is in need of treatment.

### Limitations

Clinical relevance of the findings from this study should be interpreted in the light of some shortcomings. First, the short duration of follow-up has already been mentioned. Second, the relationship between offending and externalizing disorders is not an independent one, since concepts of ODD/CD and offending overlap. True overlap may be limited in our group, though, as most individuals had ODD, which is not characterized by delinquent behavior. Second, due to limited language competencies, the assessment of psychiatric diagnoses among non-Western juveniles was constrained. Last, as has also been mentioned before, it was not feasible to distinguish between ODD and CD or between the different types of ADHD [25] as the number of participants in these subgroups was not large enough to yield sufficient power for statistical analysis.

### Clinical implications

Screening for those at risk of persistence is needed in childhood first-time police arrestees, as the majority of this group follows a relatively benign course of offending, while development of a smaller subgroup is worrisome. Screening should be sensitive to externalizing psychopathology, given its predictive value, frequent occurrence, and availability of evidence-based interventions for these disorders [44, 45].

## Appendix—Case 1: Persistent high

A. was first arrested by the police at 11 years of age, while joy-riding his father’s motorcycle in the woods. Although A.’s intelligence was in the normal range, he had been in special needs education schools since he was 9 years of age. Child psychologists had diagnosed him with “fear of failure” and “a lag in his socio-emotional development”. A.’s teacher had noticed his aggressive behavior and thought it likely that both A.’s peers and parents performed antisocial acts. A.’s mother had suffered from a depression since she divorced A.’s father 3 years ago. On the Diagnostic Interview Schedule for Children, A. met criteria for attention deficit/hyperactivity disorder as well as oppositional defiant disorder. When followed up 2 years later, A. confessed to still steal, fight, threaten other children, lie, defy authority, and drink alcoholic beverages. At the age of 15 years, A. was arrested for raping a girl with a group of friends, threatening to shoot her if she would resist.

## Case 2: Persistent low

B. was 10 years of age when he was arrested for throwing stones at windows and breaking one. B.’s mother had used alcohol during pregnancy and was battered by B.’s father, a Turkish alcoholic, whom she had never married and who left when B. was only 1 year of age. B.’s mother had a severe burnout. B., who had a total IQ of 120, went to special needs education schools, because, he never listened to his teacher in regular schools. B. had antisocial peers, cursed a lot, and was always angry, but was empathic when people were hurt. On the Diagnostic Interview Schedule for Children, B. met criteria for oppositional defiant disorder. Child psychologists had diagnosed B. with “emotional problems” as well as “low social skills”. They started play therapy and social skills training. When followed up 2 years later, B. was less angry. Besides breaking the window, B. has never committed any offense.

## Case 3: Occasional

C. was first arrested at 7 years of age, after stealing sunglasses from a shop. C. lived with his grandmother, who punished him severely for his offense. C.’s father was unknown to his Antillean mother, who was in jail herself for drug trafficking. C. and his grandmother lived in a low socio-economic status neighborhood and had financial problems. C. confessed to steal, both at home and in shops. C.’s IQ was 68 and didn’t like school: he fought and threatened other children when at school, and skipped classes now and then. On the Diagnostic Interview Schedule for Children he met criteria for attention deficit/hyperactivity disorder. When followed up two years later, C.’s mother was back at home, living with him and his grandmother. A still fought with his little brother now and then, but did not fight at school. Furthermore, he stopped stealing altogether.

## Case 4: Persistent low

D. was 5 years of age, when he and his brother were arrested for prank-calling the police. D. lived in a low socio-economic status neighborhood in Rotterdam, in a traditional Moroccan family. While D. did not meet criteria for any externalizing disorder in the Diagnostic Interview Schedule for Children, his teacher did report some externalizing symptoms: D. was a real bully, fought with other children and was hyperactive in the classroom. D.’s teacher also questioned D.’s parents’ child-rearing skills. However, no delinquency was reported. At follow up, when D. was almost 8 years of age, there was no increase in delinquent behavior. Although his teacher still qualified his family as “unsafe”, and D. as a hyperactive and oppositional kid, his bullying and fighting had diminished.

## Footnotes

1 In this paper, children detained by the police or reprimanded on the street due to illegal behavior are called arrestees. Status offenses are not included in this definition.

2 As there was some variation between participants in time between initial assessment and follow-up, analyses were also run taking into account time to follow-up, which did not lead to different results.

3 To rule out that findings on the predictive value of externalizing psychopathology were solely based on the fact that parents both reported on externalizing psychopathology and level of offending, analyses were also run using only child reports on level of offending. This did not lead to different results.
